# Cross-Condition Tool Wear State Monitoring via Multi-Source Sensor Signal Fusion and Supervised Transfer Learning

**DOI:** 10.3390/s26113423

**Published:** 2026-05-28

**Authors:** Yifeng Huang, Xikang Lu, Daode Zhang

**Affiliations:** School of Mechanical Engineering, Hubei University of Technology, Wuhan 430068, China; kang_2020413@163.com (X.L.);

**Keywords:** tool wear state monitoring, multi-source sensor signal fusion, supervised transfer learning, cross-condition recognition

## Abstract

**Highlights:**

**What are the main findings?**

- A cross-condition tool wear monitoring framework was developed by combining multi-source sensor signal fusion with supervised transfer learning.
- The fusion of X-axis vibration, Z-axis vibration, and spindle current signals achieved better recognition performance than single- or dual-channel inputs.

**What are the implications of the main findings?**

- Multi-source sensor fusion helps improve tool wear representation when monitoring signals vary across operating conditions.
- The proposed progressive supervised transfer procedure provides a practical way to improve target-condition adaptation using limited labeled target-domain data.

**Abstract:**

Tool wear state monitoring under varying operating conditions is important for machining quality and production reliability. However, changes in cutting parameters can shift monitoring-signal distributions and reduce the generalization ability of data-driven models. This paper proposes a cross-condition tool wear state monitoring method based on multi-source sensor signal fusion and supervised transfer learning. X-axis vibration, Z-axis vibration, and spindle current signals are organized as multi-channel time-series inputs. A deep model integrating a multi-scale convolutional neural network, bidirectional long short-term memory, and an attention mechanism is developed to extract discriminative wear-related features. Source-domain pretraining, target-domain warm-up fine-tuning, and source-target joint fine-tuning are organized as a progressive supervised transfer procedure to improve target-condition adaptation. Experiments are conducted on a custom multi-condition dataset using an hp0 + hp1 → hp2 transfer task. Under the unified XZI input configuration, the proposed method outperforms CNN-LSTM, DANN, and CORAL. Input ablation results show that X, XZ, and XZI achieve accuracies of 0.6000, 0.7647, and 0.8588, respectively. In repeated random-seed experiments, the method obtains an Accuracy of 0.7929 ± 0.0499, a Macro-F1 of 0.7292 ± 0.0706, and a Cohen’s Kappa of 0.6542 ± 0.0840. The results demonstrate the effectiveness of multi-source sensor fusion and supervised target-condition adaptation for cross-condition tool wear monitoring.

## 1. Introduction

Cutting tools are key components directly involved in material removal in computerized numerical control (CNC) machining, and their wear states directly affect workpiece surface quality, dimensional accuracy, production efficiency, and machine tool operational safety. During continuous machining, flank wear, edge blunting, and local tool damage gradually accumulate with cutting time. Excessive tool wear can increase cutting force, intensify vibration, enlarge machining errors, and even cause tool failure or unexpected downtime. Therefore, tool wear monitoring has become an important research topic in intelligent manufacturing, integrating sensing, modeling, and decision-making for machining process control [1].

With the development of intelligent sensors, data acquisition systems, and artificial intelligence methods, tool wear monitoring has gradually shifted from offline measurement and experience-based judgment to online sensing and data-driven modeling. Teti et al. reviewed advanced machining process monitoring techniques and indicated that sensor signals, feature extraction, and intelligent decision-making methods are essential for online machining state assessment [2]. In practical machining processes, different sensor signals can reflect tool states from different perspectives. Dimla reviewed sensor signals for tool wear monitoring in metal cutting operations and pointed out that cutting force, vibration, acoustic emission, current, and power signals can all provide useful information for tool condition monitoring [3]. Kang et al. further proposed a multi-sensor tool wear monitoring method for industrial scenarios, showing that the fusion of spindle power and vibration signals can improve the applicability of tool wear monitoring in practical machining environments [4]. These studies indicate that multi-source sensor signal fusion is an effective way to enhance tool wear state representation under complex machining conditions.

Tool wear monitoring can be implemented using different sensing principles, and each signal type reflects the machining process from a different physical perspective. Cutting force is closely related to the tool–workpiece contact state and has been widely used to monitor wear-induced changes during machining. Force/load-sensor-based monitoring has also been applied to real-time quality assessment in progressive stamping processes, showing that force variation can reflect process abnormality and quality changes [5]. In situ force-response monitoring has further been used to characterize wear-induced edge-quality degradation in trimmed steel sheets [6]. Acoustic and sound-based monitoring methods provide another practical route for online tool condition assessment. For example, sound pressure signals have been analyzed in the frequency domain for tool wear monitoring during turning processes [7]. Audio signals have also been used to monitor tool wear progression in sheet-metal stamping processes [8]. Overall, these findings show that tool wear is reflected through multiple physical responses rather than a single isolated signal, which provides the motivation for multi-source sensor signal fusion.

Deep learning has been widely used in tool wear monitoring because of its ability to automatically learn nonlinear and hierarchical features from complex signals. LeCun et al. demonstrated that deep learning can learn multi-level representations from data, which provides a theoretical basis for feature learning from complex monitoring signals [9]. Martínez-Arellano et al. transformed time-series signals into image representations and used deep learning for tool wear classification, showing that deep models can extract wear-related information from transformed signal representations [10]. Tool wear signals usually contain local transient fluctuations, periodic impacts, and long-term trends. Xu et al. introduced multi-scale feature fusion and a channel attention mechanism into tool wear prediction, indicating that multi-scale feature extraction is effective for machining signals with complex temporal patterns [11]. Liu et al. proposed a tool wear monitoring model based on parallel residual structures and stacked BiLSTM networks, demonstrating the value of temporal dependency modeling in tool wear monitoring [12]. Guo et al. used an attention-based deep learning model for high-speed milling tool wear monitoring, showing that attention mechanisms can enhance key wear-related features [13]. Recent studies have also focused on sensing generalization ability in milling tool wear monitoring, aiming to reduce performance variation among different sensing channels [14]. This line of research suggests that both feature representation ability and sensing robustness are important for practical tool wear monitoring.

However, many existing deep learning models are trained and tested under a single or relatively stable operating condition. In real manufacturing environments, cutting parameters, spindle speed, feed rate, cutting depth, tool state, and machining load may vary, causing the distributions of monitoring signals to change across operating conditions. When a model trained on source conditions is directly applied to a new target condition, its recognition performance may degrade due to domain shift. Pan and Yang systematically reviewed transfer learning and pointed out that source-domain knowledge can be used to support target-domain modeling when training and testing data do not follow the same distribution [15]. For tool wear monitoring under variable working conditions, Li et al. proposed a feature-based transfer learning method to establish feature associations between source and target domains [16]. Zhang et al. developed a tool wear monitoring method based on multi-channel hybrid information and deep transfer learning, showing the potential of combining multi-source information with transfer learning under varying operating conditions [17]. Zhou et al. integrated a depthwise separable convolutional neural network with a pretraining/fine-tuning transfer learning strategy to improve online tool wear monitoring under variable working conditions [18].

Recent deep transfer learning studies in industrial diagnosis have also explored more complex domain adaptation and robustness mechanisms. Some studies have introduced conditional distribution information into adversarial transfer learning to improve multi-source domain adaptation by reducing distribution discrepancies between domains [19]. Other studies have used spatial-channel collaborative graph interaction models to strengthen cross-domain feature interaction and transferability in industrial fault diagnosis tasks [20]. Fault-tolerant and robustness-oriented control methods have also attracted attention because practical monitoring systems may suffer from sensor noise, signal uncertainty, actuator faults, or abnormal operating disturbances [21]. These studies indicate that cross-domain industrial diagnosis is moving from simple marginal distribution alignment toward more detailed conditional, structural, multi-source, and robustness-aware feature adaptation. Compared with these unsupervised, graph-based, or fault-tolerant methods, the present study focuses on a supervised transfer learning setting in which a limited number of labeled target-domain samples are available for target-condition adaptation and model selection.

Although these studies have achieved promising results, several issues remain. First, some methods focus on single sensor signals or manually designed features, making it difficult to fully exploit the complementary information among different physical quantities such as vibration and spindle current. Second, although deep models have strong feature extraction capabilities, their performance may still degrade when source and target operating conditions differ significantly. Third, many domain adaptation methods mainly focus on unlabeled target domains, whereas in practical machining scenarios, a small number of labeled target-domain samples are often available for fine-tuning and model selection. Therefore, supervised transfer learning with limited labeled target-domain data deserves further investigation for cross-condition tool wear monitoring.

To address these issues, this study proposes a cross-condition tool wear state monitoring method based on multi-source sensor signal fusion and supervised transfer learning. X-axis vibration, Z-axis vibration, and spindle current signals are used to construct multi-channel time-series samples. A deep feature extraction model integrating a multi-scale convolutional neural network, BiLSTM, and an attention mechanism is developed to learn discriminative wear-related representations. Source-domain pretraining, target-domain warm-up fine-tuning, and source-target joint fine-tuning are organized as a progressive supervised transfer procedure to improve adaptation to the target operating condition. The proposed method is evaluated on a custom multi-condition tool wear dataset using an hp0 + hp1 → hp2 transfer task. Input ablation, transfer strategy comparison, model comparison, and multi-random-seed robustness experiments are conducted to verify the effectiveness and stability of the proposed method.

## 2. Signal Representation and Proposed Method

### 2.1. Custom Experimental Platform and Task Definition

The milling experiments were conducted on an MLV 600 three-axis machining center equipped with a FANUC Series 0i-MF CNC system (FANUC Corporation, Yamanashi, Japan). A four-flute tungsten carbide end mill with a hardness of HRC 70 was used as the cutting tool, and H13 die steel with a size of 150 mm × 100 mm × 25 mm and a hardness of HRC 20–25 was used as the workpiece material.

This study was conducted using a custom multi-condition tool wear dataset. The dataset was collected from a self-built tool machining experimental platform. All experiments were carried out on the same three-axis machining center, and on-site milling tests were performed under a unified workpiece coordinate system. To ensure geometric consistency and comparability among different batches of experimental data, the image acquisition position was fixed during the experiments, and the image acquisition device was pre-calibrated. In addition, multi-channel data acquisition and file naming were uniformly controlled by scripts, ensuring the correspondence of different modal data in both the temporal and sample dimensions.

In terms of data acquisition, vibration, cutting force, acoustic emission, machine electrical power, and images of the bottom and side edges of the tool were synchronously collected during the experiments. During data acquisition, the X-axis and Z-axis vibration signals were measured using VTall-T163E-A acceleration sensors at a sampling rate of 26.7 kS/s. Cutting force was recorded using a Yuli M4325R force sensor and an M8301A amplifier (Sunrise Instruments Co., Ltd., Nanning, China), together with an SMACQ3113 data acquisition card (Smacq Technologies Co., Ltd., Beijing, China), at a sampling rate of 12 kS/s. Spindle electrical power was measured using a TH3444 power meter (Changzhou Tonghui Electronic Co., Ltd., Changzhou, China) at a sampling rate of 10 S/s. Acoustic emission signals were collected using a Fuji AEW144S acoustic emission sensor (Fuji Ceramics Corporation, Fujinomiya, Japan) with a PXPA3 preamplifier (Changsha Pengxiang Electronic Technology Co., Ltd., Changsha, China) at a sampling rate of 2.5 MS/s. Tool images were captured using TD-KM-4KH and AO-HK810-0318 cameras with a resolution of 3840 × 2160 pixels. Considering the effectiveness of input information, the convenience of continuous acquisition, and the feasibility of engineering deployment for the subsequent state recognition model, X-axis vibration, Z-axis vibration, and spindle current signals were selected as the main inputs in this study. Compared with cutting force, acoustic emission, and image information, vibration and current signals are easier to acquire continuously during machining. In addition, they can reflect tool wear states from the perspectives of mechanical dynamic response and spindle load variation, respectively [22]. Therefore, cutting force, acoustic emission, and image data were mainly used for experimental recording and future extended research, and were not included in the main cross-condition recognition model.

In terms of operating condition settings, different operating conditions were constructed mainly by adjusting machining parameters while keeping the tool type, workpiece material, and clamping method unchanged. This design was used to investigate the relationship among machining parameters, process responses, and tool wear under different conditions. According to the experimental settings, the dataset was divided into three operating conditions, namely hp0, hp1, and hp2. The corresponding machining parameters are listed in Table 1.

It should be noted that the purpose of this study was not to cover all possible combinations of cutting parameters, but to construct a representative cross-condition recognition task with clear distribution differences among operating conditions. The three operating conditions were selected by changing spindle speed and feed rate while keeping the tool type, workpiece material, clamping method, and radial depth of cut consistent. In this way, the influence of operating-condition variation on monitoring signals could be investigated while avoiding excessive interference from unrelated experimental factors. Although the number of operating conditions is limited, the selected hp0, hp1, and hp2 settings introduce different combinations of spindle speed and feed rate and therefore lead to changes in vibration response, spindle load, and tool wear evolution. The hp0 + hp1 → hp2 task was designed to evaluate whether the model trained with source-condition information can adapt to a target operating condition with a different signal distribution. Therefore, the dataset is more suitable for validating the feasibility of supervised cross-condition transfer learning under limited experimental conditions, rather than claiming complete coverage of all machining parameter spaces.

**Figure 1:**
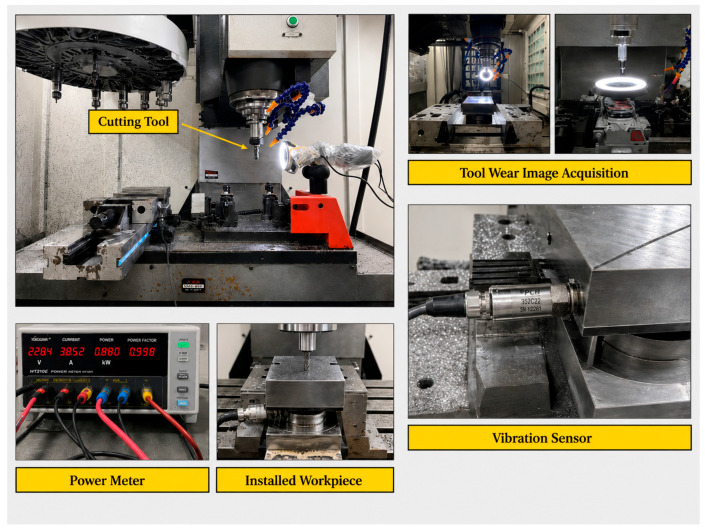
Experimental Scenario.

**Figure 2:**
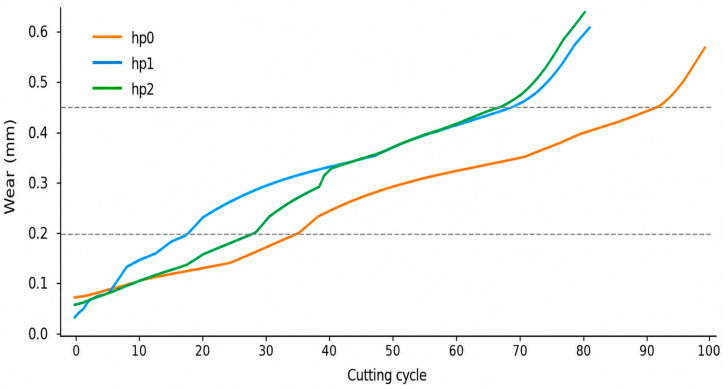
Tool wear evolution curves and wear-state thresholds under hp0, hp1, and hp2 conditions. The dashed horizontal lines indicate the wear-state thresholds of 0.20 mm and 0.45 mm.

Under each operating condition, the samples were further divided into three wear states according to the measured tool wear value, denoted as class 0, class 1, and class 2. In this study, tool wear was measured from the side-edge images of the cutting tool collected by the camera system shown in Figure 1. The wear width was obtained using image-based measurement software after spatial calibration of the tool image. The tool wear evolution curves and the corresponding wear-state division criteria are shown in Figure 2, where the three curves correspond to hp0, hp1, and hp2, respectively. It should be noted that the first point of each curve does not represent a brand-new tool before machining. Instead, the wear value was recorded after the first cutting experiment, which explains why the curves do not start from 0 mm. Since each operating condition was conducted as one continuous wear experiment, Figure 2 shows the measured wear trajectory under each condition rather than the average of repeated tests; therefore, error bars were not added. These three classes correspond to slight wear, moderate wear, and severe wear, respectively. Wear values below 0.20 mm were labeled as class 0, wear values between 0.20 mm and 0.45 mm were labeled as class 1, and wear values above 0.45 mm were labeled as class 2.

Specifically, the numbers of samples in classes 0, 1, and 2 were 245, 392, and 56 under hp0; 128, 352, and 83 under hp1; and 196, 273, and 91 under hp2, respectively. The sample distribution is summarized in Table 2. It can be observed that the class distribution within each operating condition is not completely balanced. The number of samples in the moderate wear state is relatively larger, whereas the number of samples in the severe wear state is relatively smaller. This distribution is closer to practical engineering scenarios to some extent and further increases the difficulty of cross-condition tool wear state recognition.

Based on the custom multi-condition dataset described above, a cross-condition tool wear state recognition task of hp0 + hp1 → hp2 was further constructed. In this task, hp0 and hp1 were jointly used as the source domain to learn general discriminative features of tool wear states, while hp2 was used as the target domain to evaluate the adaptation and generalization performance of the model under a new operating condition. Unlike single-condition scenarios where training and testing data generally follow similar distributions, this task explicitly introduces data distribution shifts caused by changes in operating conditions, making it more representative of practical tool wear monitoring requirements in complex manufacturing environments.

### 2.2. Multi-Source Signal Construction and Sample Organization

Multisensor data fusion has been shown to improve tool wear prediction by integrating complementary information from different monitoring channels [23]. In the cross-condition experiments, the available raw signals included X-axis vibration, Z-axis vibration, and spindle current. To ensure consistency in data format across different experimental settings, these three types of signals were first uniformly organized and then constructed into multi-channel time-series samples. Let the length of a single sample be denoted as T, and let the number of input channels be denoted as C. The input sample of the model can then be expressed as

(1) $$X\in R^{T\times C}$$

where C = 1, C = 2, and C = 3 correspond to the X, XZ, and XZI input configurations, respectively.

In terms of data partitioning, hp0 and hp1 were jointly used as the source domain, while hp2 was used as the target domain. For the source domain, samples from each source condition were stratified by class, with 80% used for training and 20% used for validation. The training subsets and validation subsets from the two source conditions were then concatenated to form the unified source-domain training set and source-domain validation set, respectively. For the target domain, a two-stage partitioning strategy was adopted. First, the target-domain samples were divided into a training candidate set and a test set at a ratio of 85%:15%. Then, the training candidate set was further divided into a target-domain fine-tuning training set and a fine-tuning validation set at a ratio of 80%:20%. Therefore, from the perspective of the overall target-domain samples, the final split was approximately 68%:17%:15%, corresponding to the fine-tuning training set, fine-tuning validation set, and final test set, respectively.

To ensure comparability among different experiments, the model comparison experiments and transfer strategy analysis were conducted using the same data partitioning protocol. In the model comparison stage, all methods used the unified XZI three-channel input to avoid interference caused by differences in input information. On this basis, input channel ablation experiments were further conducted to compare the performance of the X, XZ, and XZI configurations. This analysis was used to evaluate the influence of multi-source signal fusion on cross-condition recognition performance and to determine the preferred input configuration for the main model, since multisensor fusion has been shown to provide more tool-condition-related information in milling monitoring tasks [24].

**Figure 3:**
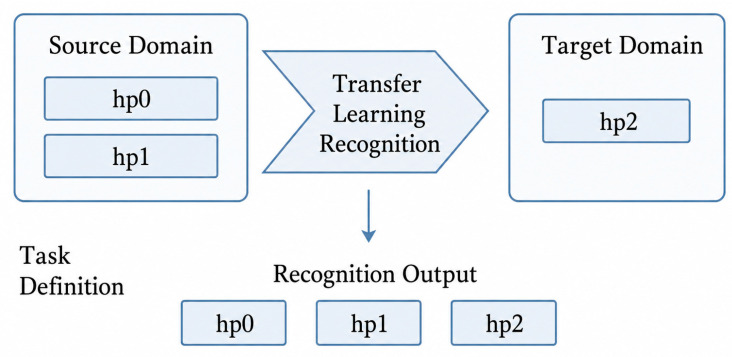
Definition of the cross-condition transfer recognition task. The arrows indicate the data organization and transfer direction from the source conditions to the target condition.

It should be noted that the target-domain training set and validation set both retained their labels and were used for model fine-tuning and parameter selection. Therefore, the cross-condition recognition task in this study belongs to a supervised transfer learning setting with labeled target-domain samples. Under this setting, the organization of multi-source signal samples should not only ensure consistency in input format, but also maintain the independence among source-domain knowledge learning, target-domain adaptation, and final test evaluation. This provides a unified and reproducible data basis for the subsequent experiments. The overall organization of the hp0 + hp1 → hp2 cross-condition transfer recognition task is shown in Figure 3.

### 2.3. Multi-Scale Convolutional Neural Network

Tool wear monitoring signals usually contain both local transient fluctuations and long-term variation trends. A single-scale convolution kernel often has difficulty effectively extracting features across different temporal scales. To enhance the representation ability of the model for multi-level wear-related information, a multi-scale convolutional neural network was used as the front-end feature extraction module in this study.

The basic idea of multi-scale convolution is to introduce convolution kernels with different sizes into the same network, thereby obtaining feature representations under different receptive fields. Smaller convolution kernels are more suitable for capturing short-term local variations and fine-grained fluctuation patterns, whereas larger convolution kernels are more effective in describing variation trends over a wider temporal range. By combining multi-scale convolution operations, the model can extract wear-related features at multiple temporal scales, thereby improving its representation ability for complex time-series signals [25].

In the implementation of the proposed model, the multi-scale convolution module was constructed using three parallel one-dimensional convolution branches. The convolution kernel sizes were set to 3, 5, and 7, respectively, and each branch contained 64 filters. The stride of each convolution branch was set to 1, and same padding was used to maintain the temporal length of the feature maps. After convolution and batch normalization, the feature maps extracted by the three branches were concatenated along the channel dimension and then fed into the subsequent BiLSTM module. This structure enables the model to extract local signal patterns under different temporal receptive fields while maintaining a consistent sequence length for temporal dependency modeling. The detailed parameter settings of the multi-scale convolution module are summarized in Table 3.

As shown in Table 3, the multi-scale convolution module consists of three parallel one-dimensional convolution branches with kernel sizes of 3, 5, and 7, respectively. From the perspective of machining signal characteristics, the convolution branch with a smaller kernel size is more sensitive to short-term local fluctuations and transient impacts, which may be related to instantaneous tool–workpiece contact changes, local vibration disturbances, or abrupt spindle load variations. The branches with larger kernel sizes cover wider temporal receptive fields and are more suitable for describing relatively continuous variation trends in vibration and current signals during tool wear evolution. Therefore, the use of convolution kernels with different receptive fields is consistent with the multi-time-scale characteristics of tool wear monitoring signals, allowing local transient responses and longer-range degradation-related variations to be jointly represented before BiLSTM-based temporal modeling.

In the proposed method, the multi-scale convolution module serves as the front-end feature extraction component. This module not only preserves key information from multi-source inputs, but also provides richer feature representations for subsequent temporal modeling and attention enhancement. Compared with a single-scale convolution structure, the parallel multi-scale convolution structure with different receptive fields is more suitable for processing multi-frequency and multi-temporal-scale signal characteristics during tool wear evolution, thus providing a more stable front-end representation for subsequent cross-condition recognition.

### 2.4. Bidirectional Long Short-Term Memory Network

After multi-scale convolutional feature extraction, a BiLSTM layer was introduced to model the temporal dependencies among local wear-related features. Compared with a unidirectional LSTM, BiLSTM uses both forward and backward contextual information, which is helpful for characterizing the sequential evolution of vibration and current signals during tool wear. Previous studies have shown that combining convolutional feature extraction with BiLSTM temporal modeling is effective for tool wear prediction [26]. In this study, BiLSTM was used to transform the local features extracted by the MSCNN module into higher-level sequence representations for subsequent attention-based feature enhancement.

### 2.5. Attention Mechanism

In this study, the attention mechanism was used after MSCNN and BiLSTM to adaptively weight the high-level temporal features. Since the extracted features may contain both wear-related information and background disturbances, attention weighting helps enhance discriminative components that are more relevant to tool wear state recognition while suppressing weakly related information. This improves the robustness of the final feature representation under cross-condition scenarios [27].

**Figure 4:**
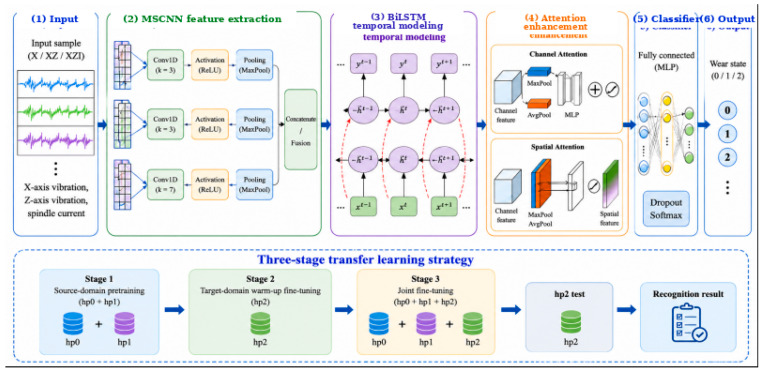
Overall framework of the proposed method. The colored boxes represent different functional modules, solid arrows indicate the main data flow, dashed arrows indicate bidirectional temporal information in the BiLSTM module, and the dashed frame denotes the progressive supervised transfer learning strategy.

In the proposed model, the attention mechanism is located at the high-level feature fusion stage. Its role is not to replace convolutional or temporal modeling, but to further select and enhance the features provided by the MSCNN and BiLSTM modules. In this way, the model can simultaneously consider local pattern extraction, temporal relationship modeling, and key feature enhancement, ultimately forming a more discriminative state representation for subsequent classification. The overall framework of the proposed method is shown in Figure 4.

### 2.6. Progressive Supervised Transfer Learning Strategy

To improve the recognition performance of the model under the target operating condition, a progressive supervised transfer strategy was adopted in this study. This strategy consists of source-domain pretraining, target-domain warm-up fine-tuning, and source-target joint fine-tuning. It follows a progressive transfer process: general features are first learned from the source domain, then preliminary adaptation to the target domain is established, and finally source-target joint optimization is performed. The purpose of this strategy is to alleviate the data distribution shift caused by changes in operating conditions [28].

In the first stage, source-domain pretraining was performed. In this stage, supervised training was performed using the source-domain training set composed of hp0 and hp1, so that the model could first learn general discriminative features related to tool wear states. Since the source domain contains a relatively larger number of samples and more complete class information, this stage mainly provides stable parameter initialization and feature representation for subsequent target-domain adaptation.

In the second stage, target-domain warm-up fine-tuning was conducted. After source-domain pretraining, the model was further fine-tuned using the target-domain training subset from hp2. In this stage, labeled samples from the target operating condition were introduced, enabling the model to gradually perceive the data distribution characteristics of the target domain while retaining the discriminative ability learned from the source domain. As a result, preliminary adaptation to the target operating condition was established.

In the third stage, source-target joint fine-tuning was performed. Different from the second-stage target-domain warm-up fine-tuning, which uses only labeled target-domain samples to adapt the model to the target operating condition, this stage jointly introduces source-domain and target-domain samples into the optimization process. The target-domain samples guide the model to adjust the decision boundary toward the target condition, whereas the source-domain samples provide additional constraints to preserve the general wear-related representations learned during source-domain pretraining. In this way, the source-domain classification objective acts as a regularization constraint during joint fine-tuning, reducing the risk that the model becomes overly biased toward the limited target-domain samples. Therefore, source-target joint fine-tuning helps alleviate catastrophic forgetting of source-domain discriminative knowledge while improving the robustness and classification stability of the model under the target operating condition.

**Figure 5:**
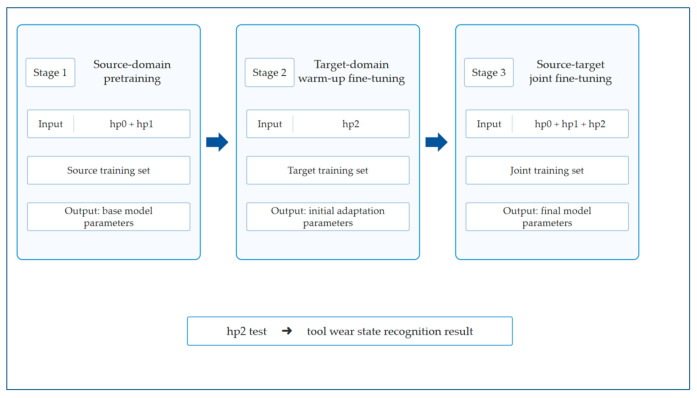
Framework of the progressive supervised transfer learning strategy. The arrows indicate the sequential training process from source-domain pretraining to target-domain warm-up fine-tuning, source-target joint fine-tuning, and target testing.

It should be noted that labeled samples were used in both the target-domain training and validation stages. Therefore, the proposed strategy is more accurately regarded as a supervised transfer learning framework rather than a strictly unsupervised domain adaptation method. Compared with source-only training or simple single-stage fine-tuning, the progressive supervised transfer strategy can make fuller use of source-domain prior knowledge and gradually enhance the adaptability of the model to the target-domain distribution. Therefore, it was adopted as the core training scheme for cross-condition tool wear state recognition in this study. The framework of the progressive supervised transfer learning strategy is shown in Figure 5.

## 3. Experimental Setup

### 3.1. Parameter Settings

To improve the reproducibility of the experimental results, the randomness of data partitioning and the randomness of training initialization were controlled separately in this study. Specifically, split_seed was used to control the partitioning of source-domain and target-domain samples, whereas train_seed was used to control network parameter initialization and other stochastic factors during model training. In the main comparison experiments, all models were evaluated using the same data partitioning protocol and training procedure to ensure fairness among different methods.

The proposed method adopted a progressive supervised transfer strategy, including source-domain pretraining, target-domain warm-up fine-tuning, and source-target joint fine-tuning. In the first stage, the model was pretrained on the source-domain training set. The learning rate was set to 1 × 10^−3^, the batch size was set to 64, and the number of training epochs was set to 50. In the second stage, warm-up fine-tuning was conducted on the target-domain training subset. The learning rate was set to 2 × 10^−4^, the batch size was set to 32, and the number of training epochs was set to 25. In the third stage, source-domain and target-domain samples were jointly used for fine-tuning. The learning rate was further reduced to 1 × 10^−5^, the batch size remained 32, and the number of training epochs was set to 20.

The model was trained using the Adam optimizer, and cross-entropy loss was adopted as the loss function. Class weights were introduced to alleviate the effect of class imbalance. During training, ReduceLROnPlateau and EarlyStopping mechanisms were used to improve training stability and reduce overfitting. The final model was selected according to the validation performance and was then evaluated on the target-domain test set. Unless otherwise stated, XZI was used as the default input configuration in the main experiments. All data processing, model training, evaluation, and visualization procedures were implemented in Python 3.10.18. The deep learning model was developed using TensorFlow 2.15.1 and Keras 2.15.0. Data processing and result organization were performed using NumPy 1.26.4 and pandas 2.3.3, metric calculation was performed using scikit-learn 1.7.2, and result visualization was performed using Matplotlib 3.10.7.

To further evaluate the overall robustness of the proposed method under different data partitions and training initializations, repeated random-seed experiments were conducted under the complete progressive supervised transfer setting [29]. Specifically, five data partition seeds, namely split_seed = 12, 22, 32, 42, and 52, and three training seeds, namely train_seed = 142, 162, and 182, were used, forming a total of 15 repeated experiments. All experiments used the same input configuration, model architecture, training procedure, and evaluation metrics, with only the data partition and training initialization being changed. Finally, the mean and standard deviation of Accuracy, Macro-F1, and Cohen’s Kappa on the target-domain test set were calculated to evaluate the robustness of the proposed method in the cross-condition recognition task.

### 3.2. Evaluation Metrics

To comprehensively evaluate the performance of the proposed model in the cross-condition tool wear state recognition task, Accuracy, Macro-F1, and Cohen’s Kappa were selected as the main evaluation metrics. In addition, confusion matrices were used to visualize the classification results. To ensure the fairness of experimental comparisons, all compared models were evaluated using the same data partitioning protocol, training procedure, and evaluation criteria.

Accuracy measures the overall proportion of correctly classified samples among all test samples. It provides an intuitive evaluation of the overall recognition performance of the model. For the multi-class classification task in this study, Accuracy is calculated based on the confusion matrix as follows:

(2) $$\mathrm{Accuracy}=\frac{\sum_{c=1}^{K}n_{\mathrm{cc}}}{N}$$

where $n_{cc}$ denotes the number of correctly classified samples of class c, K denotes the total number of classes, and N denotes the total number of test samples.

Macro-F1 calculates the F1-score for each class and then averages the F1-scores across all classes. Compared with Accuracy, Macro-F1 can more objectively reflect the recognition ability of the model for different classes, especially under imbalanced class distributions. The precision, recall, and F1-score of class c are defined as follows:

(3) $$P_{c}=\frac{TP_{c}}{TP_{c}+FP_{c}}$$

(4) $$R_{c}=\frac{TP_{c}}{TP_{c}+FN_{c}}$$

(5) $$F1_{c}=\frac{2P_{c}R_{c}}{P_{c}+R_{c}}$$

The Macro-F1 is then calculated as:

(6) $$\mathrm{Macro}-F1=\frac{1}{K}\sum_{c=1}^{K}F1_{c}$$

where $P_{c}$ and $R_{c}$ represent the precision and recall of class c, respectively. $TP_{c}$, $FP_{c}$, and $FN_{c}$ denote the true positive, false positive, and false negative samples of class c, respectively. In this study, K = 3.

Cohen’s Kappa is used to measure the agreement between the predicted results and the true labels while considering the agreement that may occur by chance. It can more effectively reflect the prediction consistency of the model in complex classification tasks. Cohen’s Kappa is defined as follows:

(7) $$\kappa =\frac{p_{0}-p_{c}}{1-p_{c}}$$

where $p_{0}$ denotes the observed agreement, and $p_{c}$ denotes the expected agreement by chance.

In addition to the above quantitative metrics, confusion matrices were further generated to visualize the classification results. In each confusion matrix, the row represents the true label and the column represents the predicted label. The value in each cell denotes the number of samples assigned to the corresponding true-predicted label pair, and the overall accuracy is marked in the title of each confusion matrix. This visualization makes it possible to more intuitively observe the classification performance of the model for different wear states and the confusion patterns among adjacent classes.

### 3.3. Compared Models and Experimental Protocol

To ensure the fairness of model comparison, XZI three-channel input was uniformly used in the main comparison experiments. CNN-LSTM, DANN, CORAL, and the proposed method were compared under the same data partitioning protocol, target-domain test set, and evaluation metrics. In this study, the CNN-LSTM, DANN, and CORAL baselines were all re-trained and evaluated under the same target-domain fine-tuning protocol. Therefore, unless otherwise specified, CNN-LSTM, DANN, and CORAL in the following model comparison refer to their fine-tuned versions under the unified experimental setting. On this basis, input channel ablation experiments were further conducted to analyze the influence of the X, XZ, and XZI input configurations on cross-condition recognition performance.

It should be noted that the cross-condition task investigated in this study is not a strictly unsupervised domain adaptation task. In the experiments, the target-domain samples were divided into a training set, a validation set, and an independent test set. The labels of the target-domain training and validation sets were used only for model fine-tuning and parameter selection, whereas the target-domain test set remained independent and was used only for final performance evaluation. Therefore, the task in this study is more accurately regarded as supervised transfer learning with limited labeled target-domain samples, rather than strictly unsupervised domain adaptation.

Under this setting, DANN and CORAL were mainly used as representative transfer learning or domain alignment baselines to compare the performance of different cross-domain modeling strategies in the current task. DANN learns domain-invariant features through a domain discriminator and a gradient reversal mechanism [30]. CORAL reduces domain discrepancy by aligning the second-order statistics of feature distributions between the source and target domains [31].

## 4. Results and Discussion

### 4.1. Comparison of Different Models Under XZI Input

Under the unified XZI input configuration, CNN-LSTM, DANN, CORAL, and the proposed method were compared on the same target-domain test set. To ensure the fairness of comparison, all models were evaluated using the same data partitioning protocol, target-domain fine-tuning protocol, target-domain test set, and evaluation metrics. The target-domain test set contained 85 samples, with 30, 41, and 14 samples in class 0, class 1, and class 2, respectively. Table 4 presents the Accuracy, Macro-F1, and Cohen’s Kappa results of different models.

As shown in Table 4, the proposed method achieved the best performance among all compared models. Its Accuracy, Macro-F1, and Cohen’s Kappa reached 0.8588, 0.8248, and 0.7675, respectively, which were clearly higher than those of CNN-LSTM, DANN, and CORAL. This indicates that, under the same input information and unified experimental protocol, the proposed method has stronger cross-condition tool wear state recognition capability.

CNN-LSTM achieved an Accuracy of 0.6471, a Macro-F1 of 0.6092, and a Cohen’s Kappa of 0.4541. Although the target-domain fine-tuning strategy enabled the model to use limited labeled target samples, its conventional convolutional and temporal modeling structure was still insufficient to fully handle the distribution shift caused by changing operating conditions. DANN improved the Accuracy to 0.6706 and the Macro-F1 to 0.6390 by introducing adversarial domain adaptation, while CORAL further increased the Accuracy, Macro-F1, and Cohen’s Kappa to 0.7059, 0.6710, and 0.5418, respectively, through feature distribution alignment. These results show that domain adaptation and fine-tuning strategies can improve target-condition recognition performance to a certain extent.

However, the performance of DANN and CORAL was still lower than that of the proposed method. This suggests that simply aligning the source and target feature distributions may not be sufficient for the current cross-condition tool wear recognition task, especially when the boundaries between adjacent wear states are difficult to distinguish and only limited target-domain samples are available. In contrast, the proposed method integrates multi-source signal fusion, multi-scale local feature extraction, temporal dependency modeling, attention-based feature enhancement, and progressive supervised target-condition adaptation into a unified framework. Therefore, it can learn more discriminative wear-related representations and better adapt them to the target operating condition.

**Figure 6:**
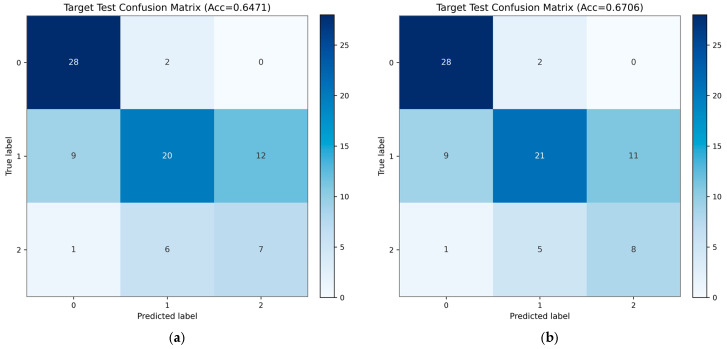

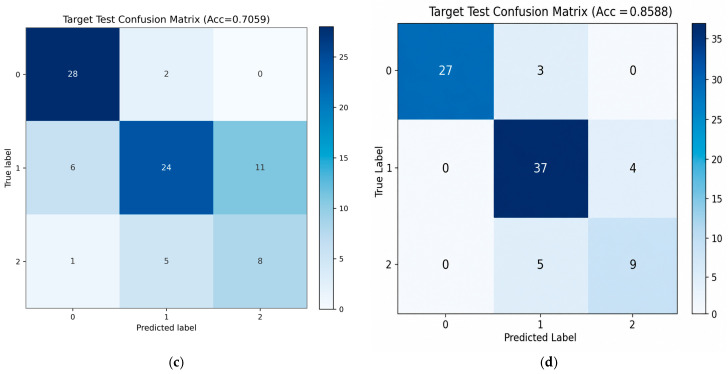
Confusion matrices of different models under the unified XZI input and target-domain fine-tuning protocol: (**a**) CNN-LSTM; (**b**) DANN; (**c**) CORAL; (**d**) the proposed method.

To further compare the classification behavior of different models for each wear state, the confusion matrices of CNN-LSTM, DANN, CORAL, and the proposed method on the target-domain test set are shown in Figure 6. As shown in Figure 6a, CNN-LSTM exhibited relatively weak discrimination ability among different wear states. Although most class 0 samples were correctly classified, a considerable number of class 1 and class 2 samples were misclassified, indicating that the model had limited ability to distinguish adjacent wear states under the target condition. Figure 6b shows that DANN improved the recognition of class 1 compared with CNN-LSTM, but misclassification between adjacent wear states still existed. Figure 6c indicates that CORAL further alleviated part of the cross-condition distribution discrepancy and achieved better overall classification results than CNN-LSTM and DANN. Nevertheless, confusion between class 1 and class 2 samples was still observed.

In comparison, the proposed method shown in Figure 6d produced clearer classification results for the three wear states. It correctly classified most class 0 and class 1 samples and maintained relatively good discrimination ability for class 2. However, a small number of class 2 samples were still misclassified as class 1, indicating that the boundary between moderate wear and severe wear remains challenging under cross-condition settings. From an engineering perspective, misclassifying severe wear as moderate wear may delay tool replacement and increase the risk of machining quality degradation or unexpected tool failure. Therefore, the recognition of severe wear should be regarded as a critical requirement in practical tool wear monitoring systems. The overall confusion among different wear states was still significantly lower than that of the compared models. This result is consistent with the quantitative metrics in Table 4 and further demonstrates the effectiveness and remaining limitations of the proposed supervised transfer strategy for cross-condition tool wear state recognition.

Overall, the results in Table 4 and Figure 6 demonstrate that, under a unified and fair experimental protocol, the proposed method not only outperformed CNN-LSTM, DANN, and CORAL in overall evaluation metrics, but also showed more balanced recognition performance across different wear states. These results indicate that the proposed method can more effectively alleviate the influence of distribution differences in cross-condition tool wear state recognition and provide a basis for the subsequent input channel ablation analysis and robustness analysis.

### 4.2. Input Channel Ablation Analysis

After the model comparison under the unified XZI input configuration, input channel ablation experiments were further conducted to analyze the influence of different signal combinations on cross-condition recognition performance. To avoid additional interference caused by differences in model structure and training strategy, only the input channel configuration was changed in this experiment, while the remaining training procedure was kept consistent. The compared input configurations included single-channel X input, dual-channel XZ input, and three-channel XZI input.

**Figure 7:**
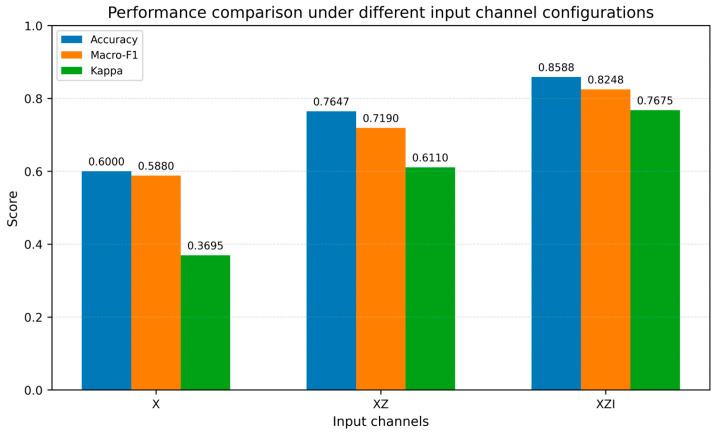
Performance comparison of the proposed method under different input channel configurations.

The quantitative results under different input channel configurations are shown in Figure 7. The experimental results indicate that the input channel configuration had a significant influence on the recognition performance. Under the single-channel X input configuration, the model achieved an Accuracy of 0.6000, a Macro-F1 of 0.5880, and a Cohen’s Kappa of 0.3695 on the target-domain test set, which were the lowest among the three configurations. This indicates that relying only on vibration signals in a single direction is insufficient to fully represent the discriminative information of tool wear states under cross-condition scenarios. When the dual-channel XZ input was used, the Accuracy, Macro-F1, and Cohen’s Kappa increased to 0.7647, 0.7190, and 0.6110, respectively. This suggests that vibration signals in two directions provide complementary information and can enhance the representation ability of the model for wear-related dynamic characteristics. After further introducing the spindle current signal, the three-channel XZI input achieved the best performance, with an Accuracy of 0.8588, a Macro-F1 of 0.8248, and a Cohen’s Kappa of 0.7675. This demonstrates that the current signal can provide effective additional discriminative information to vibration signals, thereby improving the comprehensive representation ability of the model for tool wear states.

**Figure 8:**
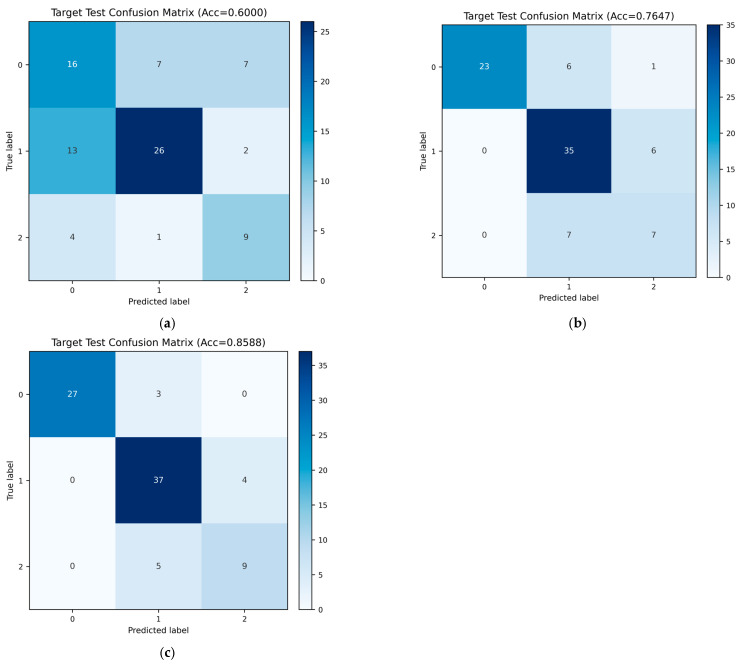
Confusion matrices under different input channel configurations: (**a**) X; (**b**) XZ; (**c**) XZI.

To further observe the classification behavior under different input configurations, the confusion matrices of X, XZ, and XZI inputs are shown in Figure 8. As shown in Figure 8a, under the X input configuration, the model exhibited obvious confusion among the three wear states, especially for adjacent wear categories. After adding the Z-axis vibration signal, the classification results became clearer, as shown in Figure 8b, indicating that dual-direction vibration input improved the separability of wear-related features. With the further introduction of spindle current, Figure 8c shows that the XZI configuration produced the most balanced classification results and the lowest overall confusion. This result is consistent with the quantitative comparison in Figure 7, further confirming the positive effect of multi-source signal fusion on cross-condition tool wear recognition.

From the overall trend, increasing the number of input channels not only improved the classification accuracy of the model, but also enhanced its stability under complex operating conditions. This indicates that the advantage of multi-source signal fusion is not merely reflected in the increase in information quantity. More importantly, the complementarity among different signal modalities helps the model obtain a more complete representation of tool wear states. Especially in cross-condition recognition tasks, a single signal is more likely to be affected by distribution shifts caused by changes in operating conditions, whereas multi-source inputs can improve the adaptability of the model to the target condition.

Therefore, XZI was adopted as the default input configuration in the subsequent main experiments. It should be noted that this conclusion was mainly obtained based on the custom dataset and the hp0 + hp1 → hp2 cross-condition recognition task in this study. Under the current experimental conditions, the joint modeling of X-axis vibration, Z-axis vibration, and spindle current can provide complementary information from the perspectives of mechanical dynamic response and spindle load variation, which is one of the important factors contributing to improved recognition performance under the target operating condition.

### 4.3. Effectiveness of the Progressive Supervised Transfer Strategy

To analyze the performance gain of the progressive supervised transfer strategy compared with source-only training, two training settings were compared in this section. In the first setting, the model was trained only using source-domain samples and then directly evaluated on the target-domain test set. In the second setting, target-domain warm-up fine-tuning and source-target joint fine-tuning were further performed after source-domain pretraining, forming the complete progressive supervised transfer procedure.

**Figure 9:**
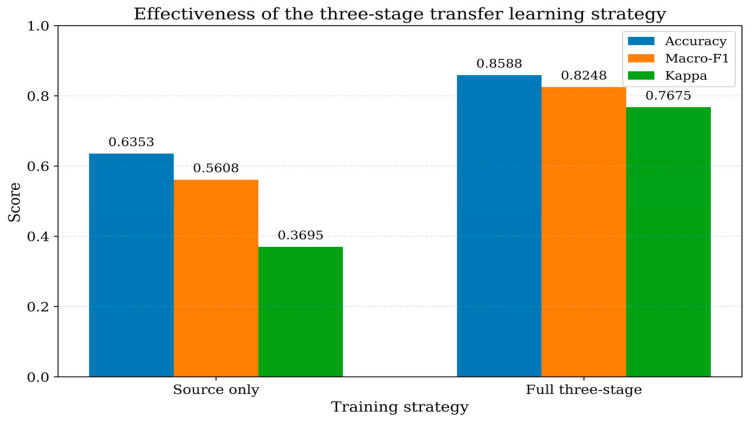
Performance comparison between source-only training and the full progressive supervised transfer strategy.

The quantitative comparison results are shown in Figure 9. Under the source-only training setting, the model achieved an Accuracy of 0.6353, a Macro-F1 of 0.5608, and a Cohen’s Kappa of 0.3695 on the target-domain test set. This indicates that the model could learn certain discriminative features related to tool wear states from the source domain. However, due to the obvious operating-condition difference between the source and target domains, its recognition performance was still greatly limited when directly transferred to the target condition. In contrast, after the complete progressive supervised transfer procedure, the Accuracy, Macro-F1, and Cohen’s Kappa increased to 0.8588, 0.8248, and 0.7675, respectively. All three metrics were significantly higher than those obtained by source-only training.

**Figure 10:**
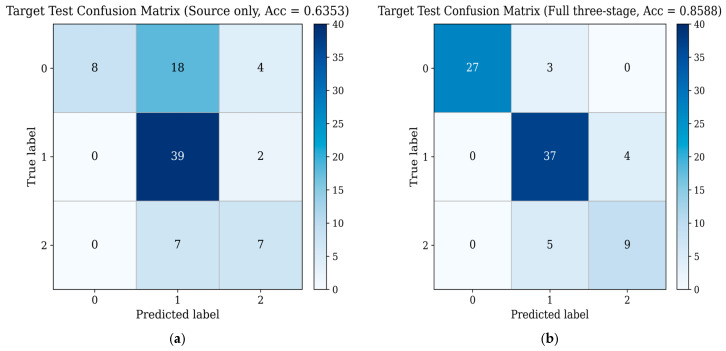
Confusion matrices under different training strategies: (**a**) source-only training; (**b**) full progressive supervised transfer strategy.

The confusion matrices under the two training strategies are shown in Figure 10. As shown in Figure 10a, source-only training produced obvious misclassification for class 0 and class 2, and many samples were incorrectly classified as the middle wear class. This suggests that the model trained only on the source domain had limited adaptability to the target-domain distribution. In comparison, Figure 10b shows that the full progressive supervised transfer strategy produced clearer classification results for different wear states. The recognition of class 0 and class 1 was substantially improved, and the confusion between adjacent wear states was reduced. These results are consistent with the quantitative metrics shown in Figure 9.

The above results indicate that source-domain pretraining can provide the model with a relatively stable initial feature representation, target-domain warm-up fine-tuning can help the model perceive the data distribution characteristics of the target condition, and source-target joint fine-tuning can further enhance target-domain adaptation while retaining source-domain discriminative knowledge. Compared with source-only training, the complete progressive supervised transfer strategy not only improved the overall classification accuracy, but also enhanced the class-balanced recognition ability and prediction consistency.

In addition, the settings of “source-domain pretraining followed by target-domain fine-tuning” and “source-domain pretraining followed by target-domain fine-tuning and source-target joint fine-tuning” were also compared. The results showed that the third-stage joint fine-tuning did not bring an additional significant single-run performance improvement under the current experimental conditions. Its main role was to maintain source-domain discriminative knowledge, reduce the risk of overfitting caused by fine-tuning on limited target-domain samples, and support a more complete progressive supervised transfer procedure. Therefore, the complete progressive supervised transfer procedure was retained as the main training scheme in this study.

Overall, the progressive supervised transfer strategy improved the recognition performance of the model under the target operating condition. Together with multi-source signal fusion and deep feature representation, it contributed to the improved cross-condition adaptation performance of the proposed method.

### 4.4. Final Cross-Condition Results of the Proposed Method

Based on the above model comparison, input channel ablation, and transfer strategy analysis, the overall robustness of the proposed method was further evaluated under the fixed main experimental setting. Specifically, under the XZI input configuration and the complete progressive supervised transfer procedure, five data partition seeds, namely split_seed = 12, 22, 32, 42, and 52, and three training seeds, namely train_seed = 142, 162, and 182, were used, forming a total of 15 repeated experiments. All experiments used the same input configuration, model architecture, and training protocol, with only the data partition and training initialization being changed.

Table 5 presents the detailed experimental results under different combinations of split_seed and train_seed. The results show that the model exhibited certain performance fluctuations under different random settings, indicating that the cross-condition tool wear state recognition task is sensitive to both data partitioning and training initialization to some extent. Among them, the results obtained under split_seed = 22 and split_seed = 52 were generally better, whereas the results under split_seed = 32 and split_seed = 42 were relatively lower. This suggests that the data partitioning strategy had a noticeable influence on the final recognition performance. From the perspective of training initialization, although different train_seed values led to performance differences under the same split_seed, the overall fluctuation remained within an acceptable range. This indicates that the proposed method has a certain degree of stability under different random initialization conditions.

To further analyze the stability of model performance from the perspective of data partitioning, the experimental results were summarized according to split_seed, and the mean and standard deviation under each partitioning condition were calculated, as shown in Table 6. It can be observed that the average performance of the model varied under different split_seed settings. Among them, the average Accuracy, Macro-F1, and Kappa values under split_seed = 22 and split_seed = 52 were relatively higher, indicating that under these partitioning conditions, the model could more effectively learn transferable relationships between the source and target domains. In contrast, the average results under split_seed = 32 and split_seed = 42 were relatively lower, further suggesting that data partitioning can influence the final performance in cross-condition recognition tasks. Overall, the standard deviations under different split_seed settings remained within a reasonable range, indicating that the performance fluctuation caused by different training initializations under the same data partition was still controllable to some extent.

On this basis, all 15 repeated experimental results were further statistically analyzed. The results show that the proposed method achieved an average Accuracy of 0.7929 ± 0.0499, an average Macro-F1 of 0.7292 ± 0.0706, and an average Cohen’s Kappa of 0.6542 ± 0.0840 on the target-domain test set. These results indicate that the proposed method can maintain relatively stable cross-condition recognition performance under different data partitioning and training initialization conditions. Compared with a single best result, the mean ± standard deviation results provide a more objective reflection of the overall performance level and robustness of the proposed method.

Based on the robustness analysis, this study further reports the representative optimal single-model result and robust ensemble result under the fixed data partitioning condition, as shown in Table 7. It should be emphasized that the mean ± standard deviation results from the repeated random-seed experiments are used to reflect the main robustness performance of the proposed method. The optimal single-model and ensemble results are mainly provided to show the relatively high performance level that the model may achieve under a fixed partitioning condition, rather than being used as the only main conclusion.

Under the fixed split_seed = 52 setting, the model achieved the best single-model result when train_seed = 162. The corresponding Accuracy, Macro-F1, and Cohen’s Kappa were 0.8588, 0.8248, and 0.7675, respectively. On this basis, models with relatively better performance were further selected to construct a robust ensemble. For the ensemble combination (142, 162, 182), the Accuracy, Macro-F1, and Cohen’s Kappa increased to 0.8706, 0.8298, and 0.7887, respectively. Compared with the optimal single model, the robust ensemble achieved improvements in all three metrics, indicating that reasonable model ensemble can reduce the influence of single-run training fluctuations to some extent and further improve the stability of cross-condition recognition results.

**Figure 11:**
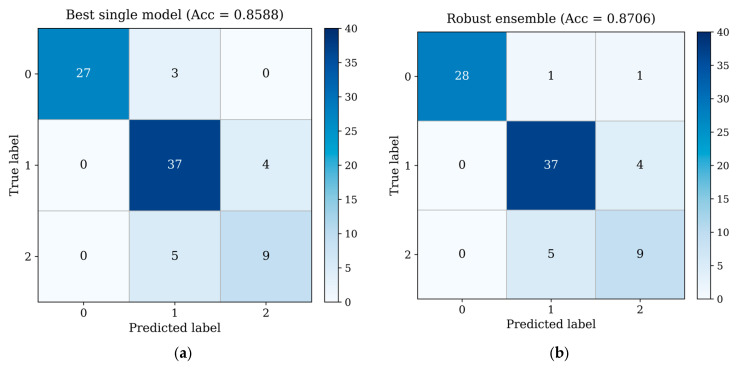
Confusion matrices of the representative fixed-partition results: (**a**) best single model; (**b**) robust ensemble.

The confusion matrices of the best single model and the robust ensemble are shown in Figure 11. Compared with the best single model, the robust ensemble further improved the recognition of class 0, reducing the misclassification of class 0 samples into class 1. The recognition results of class 1 and class 2 remained generally consistent between the two settings. This indicates that the ensemble strategy mainly improved the stability of the decision boundary for some samples while maintaining the overall classification structure learned by the best single model.

### 4.5. Discussion

The experimental results show that the combination of multi-source signal fusion and supervised target-condition adaptation can effectively improve the overall performance of the tool wear state recognition model under cross-condition scenarios. According to the input channel ablation results, the XZI configuration achieved better Accuracy, Macro-F1, and Cohen’s Kappa than the X and XZ configurations, indicating strong complementarity between vibration signals and spindle current signals. The X-axis and Z-axis vibration signals mainly reflect the mechanical response characteristics of the tool in different directions, whereas the current signal provides additional information from the perspective of spindle load and energy variation. After the three types of signals were jointly used as inputs, the model could simultaneously utilize mechanical dynamic response information and load variation information, thereby obtaining a more complete representation of tool wear states.

From the perspective of model structure, MSCNN, BiLSTM, and the attention mechanism have complementary functions. The multi-scale convolution module enhances the ability of the model to extract local patterns at different temporal scales, enabling it to capture richer local response features from raw time-series signals. BiLSTM improves the ability of the model to capture temporal dependencies, which is beneficial for characterizing the dynamic evolution of tool wear over time. The attention mechanism further strengthens the expression of key discriminative information and improves the ability of the model to suppress disturbances under complex operating conditions. Therefore, the performance improvement of the proposed method does not come from a single module, but from the joint effect of multi-source inputs, local feature extraction, temporal modeling, and key feature enhancement.

The analysis of the training strategy further indicates that the progressive supervised transfer strategy plays a positive role in target-condition adaptation. When only source-domain training was used, the model could learn certain general discriminative features, but it was difficult to directly adapt to the target-domain distribution. Through target-domain warm-up fine-tuning and source-target joint fine-tuning, the model could gradually enhance its response capability to the target domain while retaining source-domain knowledge, thereby improving cross-condition recognition performance. This suggests that, in cross-condition tool wear monitoring tasks, the design of the training strategy is as important as the design of the model structure.

Compared with domain alignment methods such as DANN and CORAL, the proposed method does not rely solely on the global alignment of feature distributions between the source and target domains. Instead, it performs supervised adaptation with a small number of labeled target-domain samples. For tool wear state recognition tasks, where class boundaries are adjacent and signal distributions vary significantly under different operating conditions, simple feature distribution alignment may not be sufficient to ensure the consistency of class-discriminative structures in the target condition. In contrast, target-domain warm-up fine-tuning can more directly adjust the model to the class boundaries of the target condition, while source-target joint fine-tuning helps improve target-domain recognition stability while preserving general discriminative knowledge learned from the source domain. Therefore, under the unified XZI input configuration, the proposed method achieved better overall performance than DANN and CORAL.

Although the proposed method achieved good experimental results on the custom multi-condition dataset, there is still room for further improvement. First, the current experiments mainly focused on three selected operating conditions, and the dataset does not cover all possible combinations of cutting speed, feed rate, and cutting depth. Future work can be extended to more cutting parameters and more complex operating environments to further evaluate the generalization ability of the model. Second, this study adopted a supervised transfer learning framework with labeled target-domain samples. In the future, transfer strategies under fewer labels or weakly supervised conditions can be considered to reduce the dependence on target-domain labels. Third, although the proposed method reduced the overall confusion among wear states, severe-wear samples may still be misclassified as moderate wear under cross-condition settings. In practical machining systems, such false-negative recognition of severe wear may delay tool replacement and increase the risk of machining quality degradation or unexpected tool failure. Therefore, future work will further consider cost-sensitive learning, decision-threshold adjustment, and safety-oriented alarm strategies to improve the reliability of severe-wear recognition. Finally, from the perspective of engineering applications, lightweight model design and online inference mechanisms can be further combined to promote the deployment of the proposed method in real-time tool wear monitoring systems.

## 5. Conclusions

To address the problem of degraded tool wear state recognition performance under cross-condition scenarios, this study proposed a cross-condition tool wear state monitoring method based on multi-source sensor signal fusion and supervised transfer learning. The proposed method used X-axis vibration, Z-axis vibration, and spindle current signals as inputs. A deep feature extraction model integrating a multi-scale convolutional neural network, bidirectional long short-term memory network, and attention mechanism was constructed. In addition, source-domain pretraining, target-domain warm-up fine-tuning, and source-target joint fine-tuning were organized as a progressive supervised transfer procedure to improve the adaptability of the model under the target operating condition. Experiments conducted on the custom multi-condition tool wear dataset showed that the proposed method outperformed CNN-LSTM, DANN, and CORAL under the unified XZI input configuration, demonstrating its strong overall performance in the cross-condition recognition task. The input channel ablation results further showed that the accuracies corresponding to the X, XZ, and XZI input configurations were 0.6000, 0.7647, and 0.8588, respectively. This indicates that the joint modeling of vibration and current signals can effectively improve the representation ability of the model for tool wear states, and XZI was the optimal input configuration under the current task setting.

In terms of robustness verification, the results of 15 repeated experiments with multiple random seeds showed that the proposed method achieved an average Accuracy of 0.7929 ± 0.0499, an average Macro-F1 of 0.7292 ± 0.0706, and an average Cohen’s Kappa of 0.6542 ± 0.0840 on the target-domain test set. These results indicate that the proposed method has good stability and target-condition adaptation ability in the hp0 + hp1 → hp2 cross-condition recognition task constructed in this study. Meanwhile, under a fixed data partitioning condition, the best single model achieved an Accuracy of 0.8588, a Macro-F1 of 0.8248, and a Cohen’s Kappa of 0.7675. After robust ensemble, these metrics were further improved to 0.8706, 0.8298, and 0.7887, respectively. These results show that the proposed method not only has good overall robustness, but also has a relatively high performance upper-bound.

Overall, the proposed framework combining multi-source signal fusion and supervised target-condition adaptation provides a technical solution with good robustness and application potential for tool wear state monitoring under multi-condition scenarios. Future work can further extend the method to more cutting parameter combinations and transfer directions. In addition, transfer modeling strategies under fewer target-domain labels and lightweight online deployment methods can be explored to promote the application of the proposed method in real-time tool wear monitoring scenarios.

## Figures and Tables

**Table 1:** Operating Conditions for a Custom-Built Tool Dataset.

Label	Feed Rate (mm/min)	Spindle Speed (rpm)	Radial Depth of Cut (mm)
hp0	600	4000	0.2
hp1	400	1600	0.2
hp2	400	4000	0.2

**Table 2:** Sample distribution of the custom-built tool wear dataset.

Condition	Class 0	Class 1	Class 2	Total
hp0	245	392	56	693
hp1	128	352	83	563
hp2	196	273	91	560

**Table 3:** Parameter settings of the multi-scale convolution module.

Convolution Branch	Kernel Size	Filters	Stride	Padding
Branch 1	3	64	1	same
Branch 2	5	64	1	same
Branch 3	7	64	1	same

**Table 4:** Performance comparison of different models under XZI input.

Model	Accuracy	Macro-F1	Cohen’s Kappa
CNN-LSTM	0.6471	0.6092	0.4541
DANN	0.6706	0.6390	0.4905
CORAL	0.7059	0.6710	0.5418
Proposed method	0.8588	0.8248	0.7675

**Table 5:** Detailed results of repeated experiments under different split_seed and train_seed combinations.

split_seed	train_seed	Accuracy	Macro-F1	Cohen’s Kappa
12	142	0.8118	0.7379	0.6798
12	162	0.7647	0.6906	0.6112
12	182	0.8118	0.7547	0.6811
22	142	0.8588	0.8279	0.7667
22	162	0.8588	0.8067	0.7641
22	182	0.8	0.7565	0.6645
32	142	0.7647	0.6346	0.5894
32	162	0.7294	0.6495	0.5562
32	182	0.7294	0.6506	0.545
42	142	0.7412	0.6752	0.5707
42	162	0.7765	0.6909	0.6174
42	182	0.7294	0.6485	0.5512
52	142	0.8	0.7733	0.6828
52	162	0.8588	0.8248	0.7675
52	182	0.8588	0.8165	0.7658

**Table 6:** Summary of repeated experimental results grouped by split_seed.

split_seed	n_runs	Accuracy	Macro-F1	Cohen’s Kappa
12	3	0.7961 ± 0.0272	0.7278 ± 0.0332	0.6574 ± 0.0400
22	3	0.8392 ± 0.0272	0.7970 ± 0.0367	0.7318 ± 0.0583
32	3	0.7412 ± 0.0204	0.6449 ± 0.0089	0.5635 ± 0.0231
42	3	0.7490 ± 0.0204	0.6715 ± 0.0214	0.5798 ± 0.0340
52	3	0.8392 ± 0.0340	0.8049 ± 0.0277	0.7387 ± 0.0484

**Table 7:** Overall robustness results and representative fixed-partition results of the proposed method.

Setting	Accuracy	Macro-F1	Cohen’s Kappa
Repeated experiments (mean ± std)	0.7929 ± 0.0499	0.7292 ± 0.0706	0.6542 ± 0.0840
split_seed = 52, train_seed = 162	0.8588	0.8248	0.7675
Robust ensemble (142, 162, 182)	0.8706	0.8298	0.7887

## Data Availability

The data supporting the findings of this study are available from the corresponding author upon reasonable request.
